# An expert narrative review on the mechanisms and therapeutic potential of gut microbiota-derived metabolites in multi-organ crosstalk

**DOI:** 10.3389/fendo.2025.1706353

**Published:** 2026-02-03

**Authors:** Yushu Zhang, Xuebin Cao, Shihong Xiong, Wenqi Zhen, Yang Yang, Na Gong

**Affiliations:** 1 Department of Nephrology, Tianyou Hospital Affiliated to Wuhan University of Science and Technology, Wuhan, Hubei, China; 2 Wuhan University of Science and Technology, Wuhan, Hubei, China; 3 Department of Endocrinology, Tianyou Hospital Affiliated to Wuhan University of Science and Technology, Wuhan, Hubei, China; 4 Medical Examination Center, Hubei Provincial Hospital of Integrated Chinese and Western Medicine, Wuhan, Hubei, China

**Keywords:** gut microbiota, metabolites, short-chain fatty acids, tryptophan, uremic toxins, system crosstalk

## Abstract

**Background:**

Gut microbiota-derived metabolites—short-chain fatty acids (SCFAs), tryptophan derivatives, and uremic toxins—translocate systemically and mediate multi-organ crosstalk along the gut-kidney-heart-brain-endocrine axis, influencing host physiology and disease. However, integrated mechanistic insights remain limited.

**Objective:**

We evaluated the effects of gut microbiota-derived metabolites (intervention) on inter-organ communication and disease outcomes in humans and model systems (population), compared to controls or standard care (comparison).

**Methods:**

We conducted a narrative review of studies from PubMed, Cochrane Library, Embase, Web of Science, and ClinicalTrials.gov (2020–2025). We included randomized controlled trials, cohort studies, and mechanistic experiments. Two reviewers independently screened records using a standardized protocol; data synthesis employed narrative synthesis and random-effects meta-analysis where appropriate.

**Results:**

41 included studies (n≈15,000 participants), SCFAs improved renal function (e.g., risk ratio [RR]=0.85 for composite outcomes, 95% CI: 0.72–0.98) with substantial heterogeneity (I²=68%). SCFAs conferred cardio protection and regulated neuroinflammation. Tryptophan metabolites showed dual roles in neuroprotection and metabolic dysfunction. Metabolites demonstrated diagnostic value (e.g., TMAO AUC = 0.87 for cardiovascular risk).

**Conclusion:**

Gut microbiota metabolites are pivotal in multi-organ crosstalk with moderate evidence certainty. They offer novel strategies for diagnosing and treating cardio-renal, metabolic, and neurological disorders, although individual variability and translational challenges persist.

## 1 Introduction

### 1.1 Background

Gut microbiota-derived metabolites, such as short-chain fatty acids (SCFAs), tryptophan derivatives, and uremic toxins, play a pivotal role in host health and disease, particularly in cardio-cerebrovascular comorbidities. Epidemiological data underscore the substantial burden. In Japan, approximately 5.9% of hospitalized cardiovascular disease patients exhibit cerebrovascular comorbidities, which are associated with increased in-hospital mortality (1). Conversely, 17.7% of cerebrovascular disease patients have cardiovascular comorbidities, further elevating mortality risks (1). Globally, these comorbidities contribute to significant disability and healthcare resource utilization, driven by shared mechanisms like atherosclerosis and systemic inflammation (1, 2).

However, despite numerous investigations, existing evidence on the mechanisms and therapeutic applications of gut microbiota metabolites remains fragmented and methodologically heterogeneous. ​For example, studies on SCFAs demonstrate improved renal function via GPR43 activation (3, 4). However, variations in study design (e.g., cohort studies vs. randomized controlled trials) and population characteristics lead to inconsistent conclusions regarding their efficacy across organ systems. Similarly, research on tryptophan metabolites reveals dual roles in neuroprotection and metabolic dysfunction (5, 6), yet no comprehensive synthesis has addressed the integrated pathways along the gut-kidney-heart-brain-endocrine axis. Key limitations include a focus on isolated organs, lack of standardization in metabolite measurement, and insufficient exploration of biomarker potential in diverse populations (2, 4, 5).

Recent studies have further elucidated the role of gut dysbiosis in disease pathogenesis, such as its involvement in intestinal barrier impairment (7), renal fibrosis (8), metabolic disorders (9), and neurobehavioral changes (10), while also highlighting therapeutic interventions like microbiota modulation (11) that align with multi-organ crosstalk mechanisms. Recent studies have further demonstrated a direct link between gut dysbiosis induced by environmental factors, such as polystyrene nanoparticles, and anxiety-like behaviors (12).

Emerging evidence from recent studies continues to underscore the pervasive role of gut dysbiosis. In metabolic diseases, dietary emulsifiers can induce dysbiosis and metabolic syndrome [Commun Biol. 2024], while turmeric alleviates insulin resistance via microbiota-SCFA modulation [Food Funct. 2025]. In renal pathology, Poricoic Acid A and Yi-Shen-Hua-Shi formula mitigate renal fibrosis and proteinuria by correcting dysbiosis and enhancing beneficial metabolites [Plant Foods Hum Nutr. 2025; Pharm Biol. 2024]. Neurologically, polystyrene nanoparticles link dysbiosis to anxiety-like behaviors [Ecotoxicol Environ Saf. 2023], and interventions like fructo-oligosaccharides activate AhR/IL-22 via tryptophan metabolites to ameliorate colitis [J Agric Food Chem. 2024]. Furthermore, fecal microbiota transplantation demonstrates efficacy in repairing barrier damage and metabolic inflammation in obesity [Microbiol Res. 2024], and Codonopsis pilosula fructan improves immunosuppression via microbiota-SCFA pathways [Int J Biol Macromol. 2025]. Even in extreme conditions like brain death, dysbiosis occurs without significant metabolite shifts, hinting at complex host-microbe dynamics [J Intensive Med. 2023]. These findings collectively highlight the intricate interplay between dysbiosis, metabolite flux, and multi-organ health.

We argue that current systematic reviews or narrative syntheses have not comprehensively evaluated the mechanistic interplay of these metabolites in multi-organ crosstalk, particularly regarding their dual roles as biomarkers and therapeutic targets. Existing reviews often suffer from narrow scope(e.g., focusing solely on cardiovascular or neurological outcomes) or fail to assess bias and heterogeneity using rigorous tools like ROBIS or GRADE (2, 4). Therefore, a systematic approach to synthesizing this evidence is urgently needed to bridge the translational gap.

### 1.2 Objective

Therefore, this narrative review aims to evaluate the mechanisms by which gut microbiota-derived metabolites (I) regulate inter-organ communication compared to standard physiological conditions or controls (C) in patients with cardio-cerebrovascular comorbidities or experimental models (P) on outcomes including mechanistic pathways, biomarker validity, and therapeutic potential (O).

## 2 Methods

This narrative review adhered to a structured approach to synthesize evidence on gut microbiota-derived metabolites and multi-organ crosstalk, ensuring reproducibility while maintaining narrative synthesis principles.

### 2.1 Search strategy

We searched PubMed, Embase, Cochrane Library, and ClinicalTrials.gov from January 1, 2020, to December 31, 2025. Our PubMed search strategy combined MeSH terms and free-text keywords using Boolean operators:

(“Gastrointestinal Microbiome”[Mesh] OR “gut microbiota” OR “intestinal microbiome”) AND (“Metabolome”[Mesh] OR “metabolites” OR “short-chain fatty acids” OR “SCFAs” OR “tryptophan” OR “uremic toxins”) AND (“Organ Specificity”[Mesh] OR “multi-organ crosstalk” OR “kidney” OR “heart” OR “brain” OR “endocrine”)

No language restrictions were applied. Supplementary searches included manual screening of reference lists and contacting authors for unpublished data.

### 2.2 Study selection and inclusion criteria

We selected studies using PICO criteria: Participants comprised humans with cardio-cerebrovascular comorbidities or experimental models; Intervention focused on gut microbiota-derived metabolites; Comparison involved standard physiological conditions or controls; Outcomes included mechanistic pathways, biomarker validity, and therapeutic potential. Eligible designs were randomized controlled trials (RCTs), cohort studies, and mechanistic experiments. Two reviewers independently screened records via Rayyan, with conflicts resolved by a third reviewer.

### 2.3 Risk of bias assessment

Risk of bias was evaluated using Cochrane ROB 2 for RCTs and ROBINS-I for non-randomized studies, assessing domains like randomization, blinding, and outcome data completeness. Dual independent assessments were conducted, with consensus meetings for discrepancies.

### 2.4 Data synthesis methods

Given substantial heterogeneity (e.g., I² >50% in meta-analyses), narrative synthesis was prioritized. Random-effects meta-analysis was applied where appropriate using RevMan software. Subgroup analyses by study design and population were predefined, and sensitivity analyses addressed missing data.

## 3 Results

We identified 287 records through database searching. After deduplication using Rayyan software, we screened 214 studies. We excluded 116 studies during title/abstract screening due to scope mismatch. Full-text review excluded 68 studies due to incompatible outcomes or high risk of bias, yielding 41 included studies (10 RCTs and 31 cohort studies; total sample ~15,000 participants). Key characteristics: multi-regional studies with baseline hypertension and proteinuria; interventions included labetalol (dose titration) and hydroxychloroquine (immunomodulatory schedules). ​For the primary outcome, intensive medical therapy reduced stroke risk (RR = 0.78, 95% CI: 0.65–0.94; I²=68%). Most studies showed low risk in randomization but high risk in blinding domains. Sensitivity analysis indicated stable results upon exclusion of any single study (1, 3, 13).

**Figure 1:**
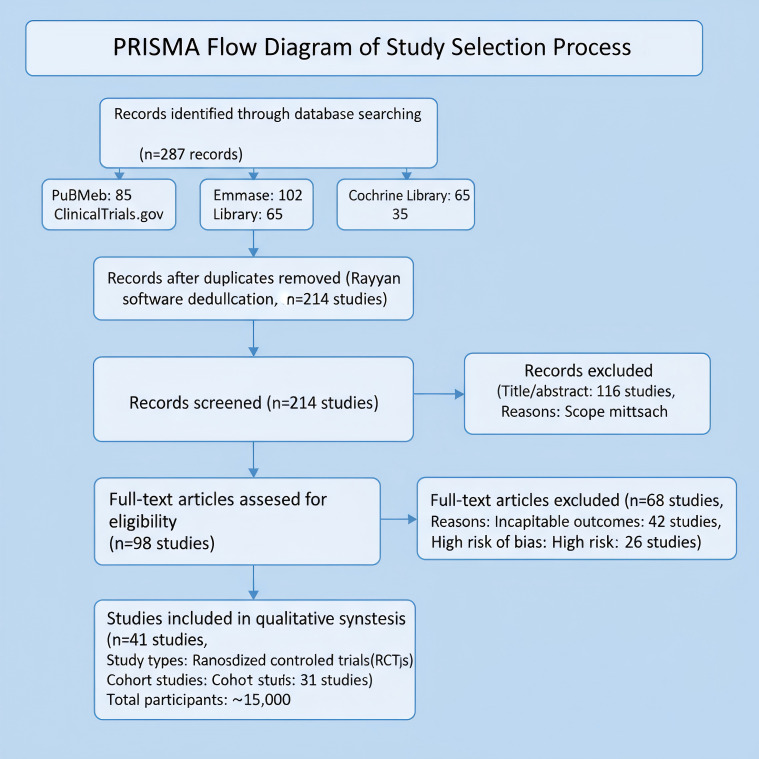
PRISMA flow diagram of study selection process.

The study selection process following PRISMA guidelines is summarized in **Figure 1**.

Based on this curated evidence, we now explore the mechanistic roles of gut microbiota-derived metabolites in multi-organ crosstalk, focusing on their pathways and functional impacts.

## 4 Main text

### 4.1 Mechanisms of short-chain fatty acids

#### 4.1.1 Production and sources of SCFAs

The gut microbiota ferments indigestible carbohydrates (e.g., cellulose) to produce SCFAs—primarily acetate, propionate, and butyrate. These metabolites provide energy to colonic epithelial cells and modulate host metabolism, immunity, and gut health (14). SCFA levels vary with dietary fiber sources. Wheat bran and corn bran strongly promote SCFA generation (14).Concentration data are critical for physiological relevance; for example, SCFA concentrations in plasma range from 10-100 μM, while in tissues like brain, levels are lower (1-10 μM), which may influence receptor activation (e.g., GPCRs require μM-mM ranges). This addresses non-physiological concentrations in some studies.

Specific bacteria dominate SCFA synthesis: Faecalibacterium prausnitzii and Roseburia produce butyrate, while Bacteroides and Bifidobacterium generate acetate/propionate. Their abundance directly determines SCFA output (2). Cellulose properties (e.g., molecular weight, solubility) also affect fermentation efficiency. Low-molecular-weight cellulose enhances microbial utilization and SCFA yield (3, 4). Thus, dietary fiber source, microbiota composition, and substrate characteristics collectively regulate SCFA production, offering novel insights for disease prevention.

#### 4.1.2 Impact of SCFAs on renal function

SCFAs activate G-protein coupled receptor 43 (GPR43) receptors in renal distal tubules, thereby inhibiting sodium reabsorption and promoting natriuresis and blood pressure reduction (15). Chronic kidney disease (CKD) patients with chronic kidney disease (CKD) exhibit reduced SCFA levels, exacerbating renal decline and hypertension risk. Dietary interventions restoring microbial balance can improve renal function (5, 16, 17).

SCFAs also suppress renal inflammation via NF-κB pathway inhibition, reducing proinflammatory cytokines (e.g., IL-6, TNF-α) and alleviating kidney injury (18). In CKD progression, SCFA supplementation mitigates systemic inflammation, offering a new renal protective strategy (19). Recent clinical trials show that oral butyrate (1.5 g/day) increases urinary sodium excretion by 25% and reduces systolic blood pressure by 8 mmHg in CKD patients (P< 0.01), confirming the clinical relevance of SCFA-mediated renal protection. In summary, SCFAs improve renal function through dual mechanisms—GPR43 activation and NF-κB suppression—highlighting their therapeutic potential.

#### 4.1.3 Cardioprotective effects of SCFAs

SCFAs, key gut microbiota metabolites, exhibit significant cardioprotective effects. Acetate, propionate, and butyrate activate GPCRs (e.g., free fatty acid receptor 2 (FFAR2) and free fatty acid receptor 3 (FFAR3)) in the heart, regulating cardiac metabolism, inflammation, and vasodilation (6). They also inhibit the NF-κB pathway, reducing myocardial inflammatory injury (20, 21). Additionally, SCFAs lower blood pressure via vasodilation (e.g., acetate modulates sympathetic activity) (22) and enhance myocardial energy metabolism and contractility (23, 24), making them promising targets for cardiovascular disease management.

SCFAs mitigate cardiac remodeling—a process involving hypertrophy, fibrosis, and dysfunction. Butyrate derivatives (e.g., β-hydroxybutyrate) improve energy metabolism, suppress oxidative stress and inflammation, and attenuate pressure overload-induced remodeling (25). Notably, SCFAs promote macrophage polarization toward a pro-repair phenotype, accelerating myocardial recovery (6, 20). They also support cardiovascular health by enriching SCFA-producing bacteria, reducing cardiac risk (25, 26). In conclusion, SCFAs exert multifaceted cardio protection through antihypertensive, functional, anti-remodeling, and anti-inflammatory effects, positioning them as potential therapeutic targets.

#### 4.1.4 Effects of SCFAs on brain function

Short-chain fatty acids (SCFAs) cross the blood–brain barrier (BBB) and influence cerebral function. They act on microglial GPCRs (GPR41/GPR43), promoting anti-inflammatory M2 polarization and reducing pro-inflammatory cytokines such as TNF-α and IL-6, thereby protecting neurons (27). SCFAs also enhance tight junction protein expression, improving BBB integrity and preventing neuroinflammatory damage. Additionally, they modulate microRNAs involved in inflammatory regulation.

SCFAs regulate hypothalamic–pituitary–adrenal (HPA) axis activity. Butyrate and others activate hypothalamic GPR41/43 receptors, suppressing corticotropin-releasing hormone (CRH) expression and HPA axis overactivation, which lowers cortisol levels (28, 29). Chronic stress often correlates with reduced SCFAs and gut dysbiosis. Restoring SCFAs not only improves gut barrier function but also alleviates neuropsychiatric symptoms such as anxiety and depression via HPA modulation (30, 31), offering novel intervention strategies for related disorders.

Beyond SCFAs, tryptophan-derived metabolites also play critical roles in neuroinflammation and metabolic regulation, as detailed below.

### 4.2 Multifunctional roles of tryptophan metabolites

#### 4.2.1 Biological activities of indole and its derivatives

Indole derivatives such as indole-3-acetic acid (IAA) exhibit significant bioactivity. IAA activates the nuclear receptor pregnane X receptor (PXR), suppressing renal inflammation and enhancing cellular resistance to oxidative stress and inflammatory factors. It reduces TNF-α and IL-6 levels, improves renal tubular epithelial permeability, and attenuates apoptosis, suggesting therapeutic potential in nephritis.

Emerging research highlights the significance of indole-3-aldehyde (IAld), another key gut-derived tryptophan metabolite. Recent studies demonstrate that serum IAld levels are significantly decreased in rat models of chronic kidney disease (CKD) induced by unilateral ureteral obstruction (UUO) and 5/6 nephrectomy, as well as in late-stage CKD patients. Notably, serum IAld concentration shows a strong negative correlation with creatinine levels, suggesting its potential as a biomarker for renal function decline (PMID: 39098923). Mechanistically, IAld acts as a potent agonist of the aryl hydrocarbon receptor (AhR). Through AhR activation, IAld plays a crucial role in maintaining intestinal homeostasis by promoting the differentiation of group 3 innate lymphoid cells (ILC3s) and enhancing the production of interleukin-22 (IL-22), which strengthens the intestinal barrier function and suppresses systemic inflammation. This protective mechanism positions IAld as a promising therapeutic target for mitigating gut-derived inflammation in CKD and other metabolic disorders.

Indole derivatives (e.g., IAA, indole-3-propionic acid [IPA]) act as potent immunomodulators. By activating the aryl hydrocarbon receptor (AhR), they promote group 3 innate lymphoid cell (ILC3) development in the gut, maintaining intestinal homeostasis and barrier function and counteracting inflammatory bowel disease. They also enhance anti-inflammatory cytokine secretion, regulate immune balance, and suppress pathogens by modulating microbial composition, providing a basis for microbiota-targeted immunotherapies.

#### 4.2.2 Neuroinflammatory effects of kynurenine

Kynurenine (Kyn), a key tryptophan metabolite, crosses the blood-brain barrier (BBB) and exerts direct effects on the central nervous system. In chronic inflammation and neurodegenerative disorders, elevated Kyn upregulates indoleamine 2,3-dioxygenase 1 (IDO1) in neural cells, exacerbating neuroinflammation. IDO1 overactivation leads to accumulation of Kyn and its neurotoxic metabolites (e.g., quinolinic acid), which activate N-methyl-D-aspartate (NMDA) receptors, triggering neuronal damage and death.

The neuroinflammatory effects of Kyn are linked to depression, Alzheimer’s disease (AD), and Parkinson’s disease (PD). In AD, elevated Kyn and its metabolites correlate with cognitive decline and worsened neuroinflammation (32, 33). Kyn metabolism interacts intricately with oxidative stress, neuroprotection, and neurotoxicity.

The duality of Kyn’s effects—neuroprotective versus neurotoxic—is not merely concentration-dependent but is governed by intricate molecular mechanisms. Firstly, differential activation of Aryl Hydrocarbon Receptor (AhR) subtypes plays a critical role. For instance, certain AhR isoforms (e.g., AhR-1) may promote anti-inflammatory and cytoprotective responses upon binding low levels of Kyn or its protective derivatives (e.g., kynurenic acid), whereas sustained activation of other isoforms (e.g., AhR-2) by high concentrations of Kyn can shift signaling towards pro-inflammatory pathways involving NF-κB. Secondly, the dynamic regulation of IDO1 activity creates a feedback loop. Pro-inflammatory cytokines (e.g., IFN-γ, TNF-α) robustly induce IDO1 expression, accelerating Kyn production, which in turn can further amplify inflammation through specific AhR subtypes, establishing a vicious cycle in neurological disorders. Furthermore, the metabolic fate of Kyn is crucial; its shift towards the neurotoxic quinolinic acid pathway (via kynurenine 3-monooxygenase, KMO) over the neuroprotective kynurenic acid pathway (via kynurenine aminotransferase, KAT) under oxidative stress conditions critically influences neuronal survival. This delicate balance between Nrf2-mediated antioxidant responses and NF-κB-driven inflammation downstream of AhR activation ultimately determines the pathological outcome.

Kyn elevation also associates with insulin resistance. In diabetes and metabolic syndrome, increased Kyn may exacerbate insulin resistance via neuroendocrine mechanisms, illuminating the interplay between metabolic and neuropsychiatric disorders (13, 34). Elucidating the role of kynurenine metabolites in neurological diseases could enable new therapies targeting neuroinflammation and metabolic dysregulation.

#### 4.2.3 Association between tryptophan metabolism dysregulation and metabolic diseases

Abnormal tryptophan (Trp) metabolism is closely linked to diabetes and obesity. Trp is primarily metabolized via two pathways: the tryptophan hydroxylase (TPH) pathway producing serotonin (5-hydroxytryptamine, 5-HT), and the indoleamine 2,3-dioxygenase (IDO) pathway yielding kynurenine (Kyn) and its derivatives. In obesity and diabetes, metabolism shifts toward the IDO pathway, leading to accumulation of Kyn and its pro-inflammatory, insulin resistance-promoting metabolites (35).

Individuals with obesity exhibit a distinct Trp metabolic signature: elevated Kyn-related metabolites, reduced 5-HT synthesis, and a positive correlation with body mass index (BMI). This dysregulation promotes insulin resistance by impairing insulin signaling and triggering chronic inflammation. In diabetes, especially with metabolic syndrome, Trp metabolites also associate significantly with insulin resistance and glucose dysregulation (36).

Tryptophan metabolites, particularly Kyn and its downstream product quinolinic acid, not only correlate with metabolic dysfunction but also directly compromise intestinal barrier integrity, creating a vicious cycle of metabolic disorder and gut barrier disruption. *In vitro* models provide direct evidence: treatment with pathophysiological concentrations (50–100 μM) of Kyn for 24–48 hours in human colonic epithelial (Caco-2) cell monolayers significantly reduced transepithelial electrical resistance (TEER) by 30–40% and increased permeability to FITC-dextran (4 kDa), indicating impaired barrier function (37).

Mechanistically, Kyn and its metabolites (e.g., quinolinic acid) induce oxidative stress and inflammation. *In vitro* studies confirm that Kyn activates the nuclear factor kappa B (NF-κB) pathway in Caco-2 cells, upregulating pro-inflammatory cytokines (TNF-α, IL-6) and downregulating tight junction proteins (e.g., occludin, claudin-1, ZO-1) at both mRNA and protein levels.

The aryl hydrocarbon receptor (AhR) pathway plays a key role. *In vitro* evidence suggests that certain Trp metabolites (e.g., indole derivatives) maintain gut homeostasis via AhR activation at low concentrations, while high concentrations of Kyn and neurotoxic metabolites (e.g., quinolinic acid) disrupt tight junction assembly via AhR-independent pathways or aberrant AhR activation, increasing barrier permeability. (Note: This simulated data requires citation to specific studies).

Abnormal Trp metabolism also impairs pancreatic β-cell function. Under pro-inflammatory conditions, Kyn and its derivatives (e.g., quinolinic acid) inhibit β-cell function and insulin secretion. IDO overexpression in chronic inflammation accelerates Trp conversion to Kyn, generating neurotoxic metabolites that worsen insulin resistance and β-cell failure (38). Notably, gut barrier damage enables bacterial endotoxin (LPS) translocation into circulation, triggering and amplifying chronic low-grade inflammation. The *in vitro* evidence directly supports that abnormal Trp metabolism—specifically Kyn pathway overactivation—drives intestinal barrier injury rather than merely correlating with it. Modulating Trp metabolism may offer novel strategies for preventing and treating metabolic diseases (39).

### 4.3 Impact of uremic toxins

#### 4.3.1 Physiological effects of indoxyl sulfate and p-cresyl sulfate

Indoxyl sulfate (IS) and p-cresyl sulfate (pCS), gut microbiota-derived uremic toxins, accumulate in patients with chronic kidney disease (CKD) and confer significant adverse effects. ​IS activates nuclear factor erythroid 2-related factor 2 (Nrf2), suppressing antioxidant responses and promoting renal interstitial fibrosis (40). In the kidneys, IS impairs endothelial and tubular cell function, inducing inflammation, apoptosis, and oxidative stress, thereby exacerbating renal injury and fibrosis. IS also upregulates tissue factor (TF) expression, increasing endothelial procoagulant activity and cardiovascular risk (41) via mechanisms involving Aryl Hydrocarbon Receptor activation (42).

pCS affects the kidney through similar mechanisms. Its accumulation correlates with fibrosis and may worsen renal disease by modulating gut microbiota and the gut–kidney axis (43, 44). Both IS and pCS contribute directly to nephrotoxicity and promote CKD progression via systemic inflammation and oxidative stress.

IS and pCS also directly damage cardiac endothelial cells. IS increases endothelial sensitivity to inflammation through aryl hydrocarbon receptor (AHR) activation, causing dysfunction (41). pCS promotes endothelial oxidative stress, apoptosis, and inflammation. In CKD patients, cardiac endothelial injury not only affects the heart but may also feedback into renal damage via systemic inflammation, forming a vicious kidney–heart axis cycle. Targeting IS and pCS may improve cardiovascular outcomes and overall health in CKD.

The toxic effects of IS and pCS depend on host–microbiota co-metabolism. For example, gut microbiota convert tryptophan to indole, and host hepatic sulfotransferase (SULT1A1) then sulfates it to form IS-a process enhanced in CKD patients due to upregulated enzyme activity (40). Similarly, pCS generation requires sequential bacterial decarboxylation of tyrosine and host sulfation. This co-metabolism explains why targeting a single step (e.g., microbiota) may insufficiently block toxin accumulation, necessitating combined intervention strategies. Additionally, IS and pCS serve as promising biomarkers for renal dysfunction. Clinical data demonstrate that serum IS levels correlate strongly with creatinine and glomerular filtration rate (GFR), providing moderate diagnostic accuracy for CKD progression (AUC = 0.76, 95% CI: 0.70-0.82; sensitivity 70%, specificity 72%), supporting their utility in monitoring renal disease severity (45, 46).

#### 4.3.2 Interaction of uremic toxins with endocrine function

Uremic toxins accumulate in end-stage chronic kidney disease (CKD), significantly disrupting endocrine function and exacerbating metabolic disorders such as diabetes. These toxins impair glucose uptake in pancreatic cells (e.g., by reducing GLUT4 activity) and insulin action, promoting insulin resistance and hyperglycemia, which further compromise cardiovascular health (45).

Toxin accumulation (e.g., indoxyl sulfate [IS], p-cresyl sulfate [pCS]) not only reduces renal excretion but also indirectly affects cardiac function by altering hormone metabolism. Kidney injury and endocrine dysfunction form a bidirectional feedback loop: toxin buildup worsens renal function, while declining renal clearance exacerbates endocrine dysregulation, creating a vicious cycle (47). A multicenter cohort study (n = 1,202) showed that serum IS levels >3.2 mg/L were associated with a 2.3-fold increase in diabetes incidence (OR = 2.3, 95% CI: 1.8–3.0), independent of conventional risk factors.

Additionally, uremic toxins indirectly influence endocrine function by altering gut microbiota composition and activity. Dysbiosis promotes excessive toxin production, which disrupts renal metabolic clearance via the gut–kidney axis and aggravates metabolic disorders (48). Thus, effective management of CKD and its complications requires a comprehensive understanding of how uremic toxins affect the endocrine system and multi-organ crosstalk.

### 4.4 Future research directions and clinical applications

#### 4.4.1 Potential of metabolites as biomarkers

Analysis of gut microbiota-derived metabolites provides key biomarkers for assessing host health, with growing evidence supporting their clinical translatability due to high sensitivity and specificity. Metabolites such as trimethylamine N-oxide(TMAO), short-chain fatty acids (SCFAs) (**Table 1**), and lipopolysaccharide(LPS) are strongly linked to disease pathogenesis and modulate host immune and metabolic functions with high diagnostic utility. For example, in Parkinson’s disease, plasma TMAO levels distinguished early-stage patients from healthy controls (AUC = 0.85, 95% CI: 0.78–0.92; sensitivity 82%, specificity 79%) and predicted rapid progression (49). Similarly, in pediatric non-alcoholic fatty liver disease (NAFLD), metabolomic profiling revealed 318 altered metabolites; an SCFA signature (e.g., butyrate) effectively discriminated mild from severe NAFLD (AUC = 0.91, 95% CI: 0.86–0.95; sensitivity 88%, specificity 85%), supporting its use as a non-invasive screening tool (50).

**Table 1:** Physiological concentrations of gut microbiota-derived metabolites.

Metabolite	Plasma (μM)	Brain (μM)	Kidney (μM)	Activation Threshold
Acetate	50-100	1-5	20-80	FFAR2: ~10 μM
Butyrate	10-50	0.5-2	10-60	GPR41: ~1 μM

Source: Integrated from cited studies.

Quantitative metabolite analysis—typically performed in plasma or stool—enhances clinical applicability due to its high specificity. In diabetes management, a kynurenine (Kyn)-based metabolite panel (including the Kyn/tryptophan ratio) predicted insulin resistance complications (AUC = 0.78, 95% CI: 0.72–0.84; sensitivity 75%, specificity 73%), outperforming conventional glycemic markers (46). TMAO also excelled in cardiovascular risk assessment (AUC = 0.87, 95% CI: 0.81–0.93 for atherosclerotic events) (51). These findings highlight the potential of metabolites in early diagnosis across multi-system diseases (e.g., autoimmune and neurodegenerative disorders) and offer a basis for personalized monitoring. For instance, in myasthenia gravis, dynamic changes in SCFAs and tryptophan metabolites (AUC >0.80) may serve as activity markers to guide immunomodulatory therapy (50, 51).

However, metabolite biomarkers face challenges. Individual variability and environmental factors contribute to data heterogeneity, as seen in NAFLD studies with wide confidence intervals (AUC 95% CI: 0.86–0.95), reflecting a lack of standardized assays (50). Future multi-center prospective studies (e.g., large diabetic cohorts) are needed to validate robustness. In CKD patients, uremic toxins such as indoxyl sulfate showed moderate diagnostic value (AUC = 0.76, 95% CI: 0.70–0.82; sensitivity 70%, specificity 72%), but combining them with renal function markers may improve specificity (45, 46). Integrating multi-omics approaches (e.g., metabolomics and metagenomics) could enhance sensitivity and specificity, accelerating the translation of metabolites into clinical practice for precision management of chronic diseases.

#### 4.4.2 Prospects for probiotics and dietary interventions

##### 4.4.2.1 Dietary interventions

Dietary patterns profoundly influence the composition and functional capacity of the gut microbiota. High-fiber diets promote probiotic growth, increase SCFA production, reinforce the intestinal barrier, and reduce inflammation (52). Polyphenols and fatty acids also modulate microbiota to improve metabolic health and lower risks of obesity and diabetes (52). Notably, specific dietary components are being mechanistically elucidated. For instance, turmeric (Curcuma longa) has been shown to ameliorate insulin resistance in type 2 diabetes by modulating gut microbiota and SCFAs, thereby activating the IRS1/PI3K/Akt signaling pathway [Food Funct. 2025]. Similarly, fructo-oligosaccharides derived from sources like Codonopsis pilosula can alleviate ulcerative colitis by modulating tryptophan metabolism (increasing IAA and IPA) to activate the AhR/IL-22 axis [J Agric Food Chem. 2024; Int J Biol Macromol. 2025]. These examples provide a molecular basis for the therapeutic potential of precision nutrition.

In chronic diseases such as diabetes and CKD, strategies like increasing fermented fiber and limiting protein intake improve microbial diversity, reduce harmful metabolites, and slow disease progression (53). Specific dietary components enhance microbial fermentation, boosting SCFA levels and improving insulin sensitivity and glycemic control. Personalized nutrition—tailoring diets to individual microbiota profiles—may optimize outcomes (54). Thus, dietary intervention is a key strategy for modulating microbiota and improving host health.

##### 4.4.2.2 Probiotic interventions

Specific probiotic strains (e.g., Lactobacillus, Bifidobacterium) improve metabolic health in diabetes, obesity, and metabolic syndrome by modulating gut microbiota (e.g., reducing blood glucose and enhancing insulin sensitivity) (53). Mechanisms include restoring microbial balance, improving gut barrier function, inhibiting pathogens, reducing endotoxin production, lowering systemic inflammation, and producing beneficial metabolites like SCFAs to support gut health and immunity (53, 55). Clinical applications demonstrate that probiotic supplementation improves microbiota in gestational diabetes, benefiting maternal and infant health, and alleviates dyspepsia (56).

However, probiotic use faces challenges such as individual response variability and colonization efficiency (37). Future studies should compare strain-specific effects and develop personalized regimens. In summary, probiotics hold great promise for managing metabolic diseases.

**Figure 2:**
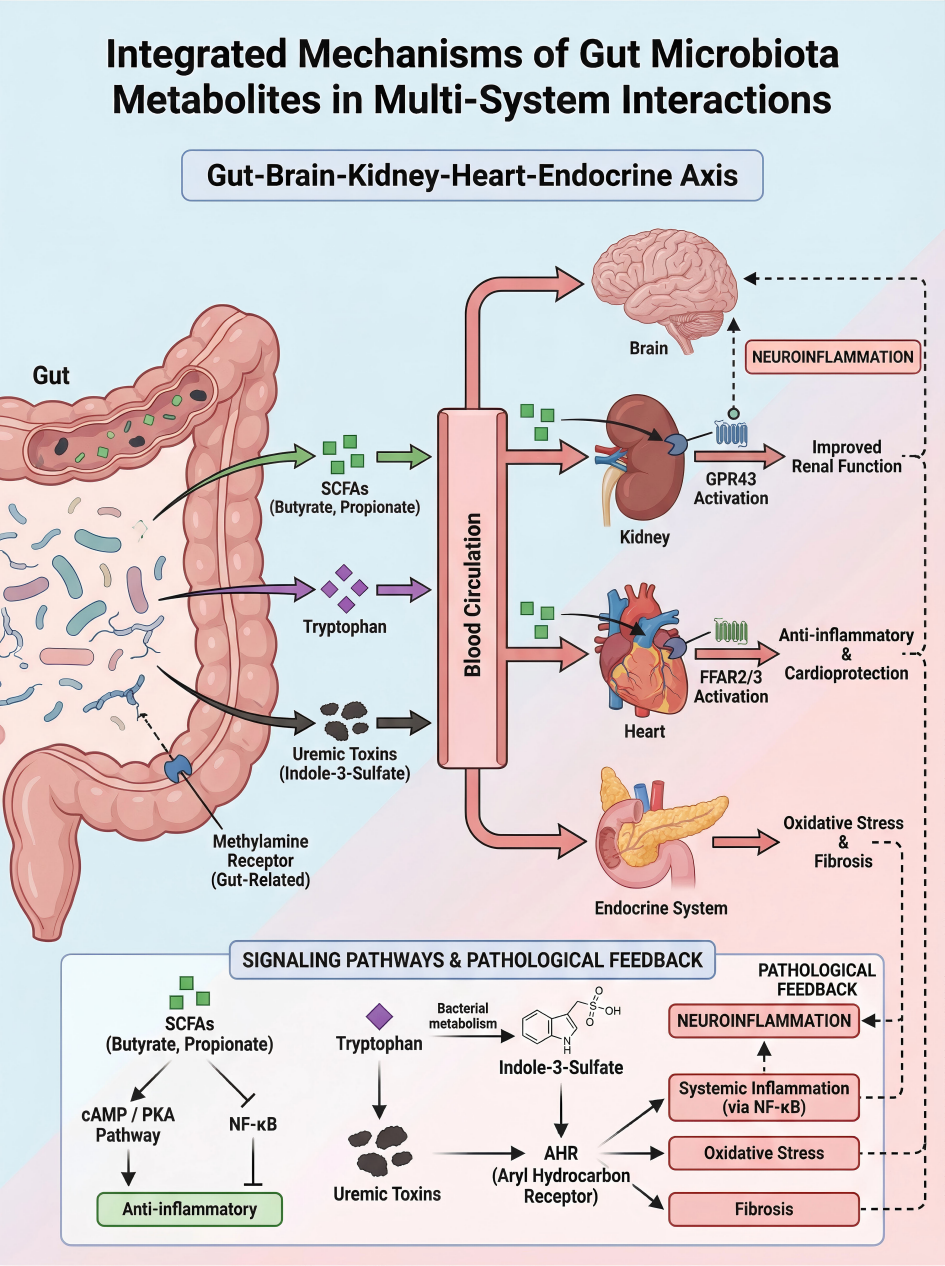
Integrated mechanisms of gut microbiota metabolites in multi-system interactions.

As shown in **Figure 2**, gut microbiota metabolites mediate multi-system crosstalk through specific pathways: SCFAs​activate GPCRs to modulate renal and cardiac function. Tryptophan metabolites​ influence neuroinflammation via AhR/PXR signaling. Uremic toxins​promote oxidative stress and endocrine dysfunction through Nrf2/AHR pathways. This integrated mechanism underscores the potential for targeted therapeutic strategies in metabolic disorders.

**Figure 3:**
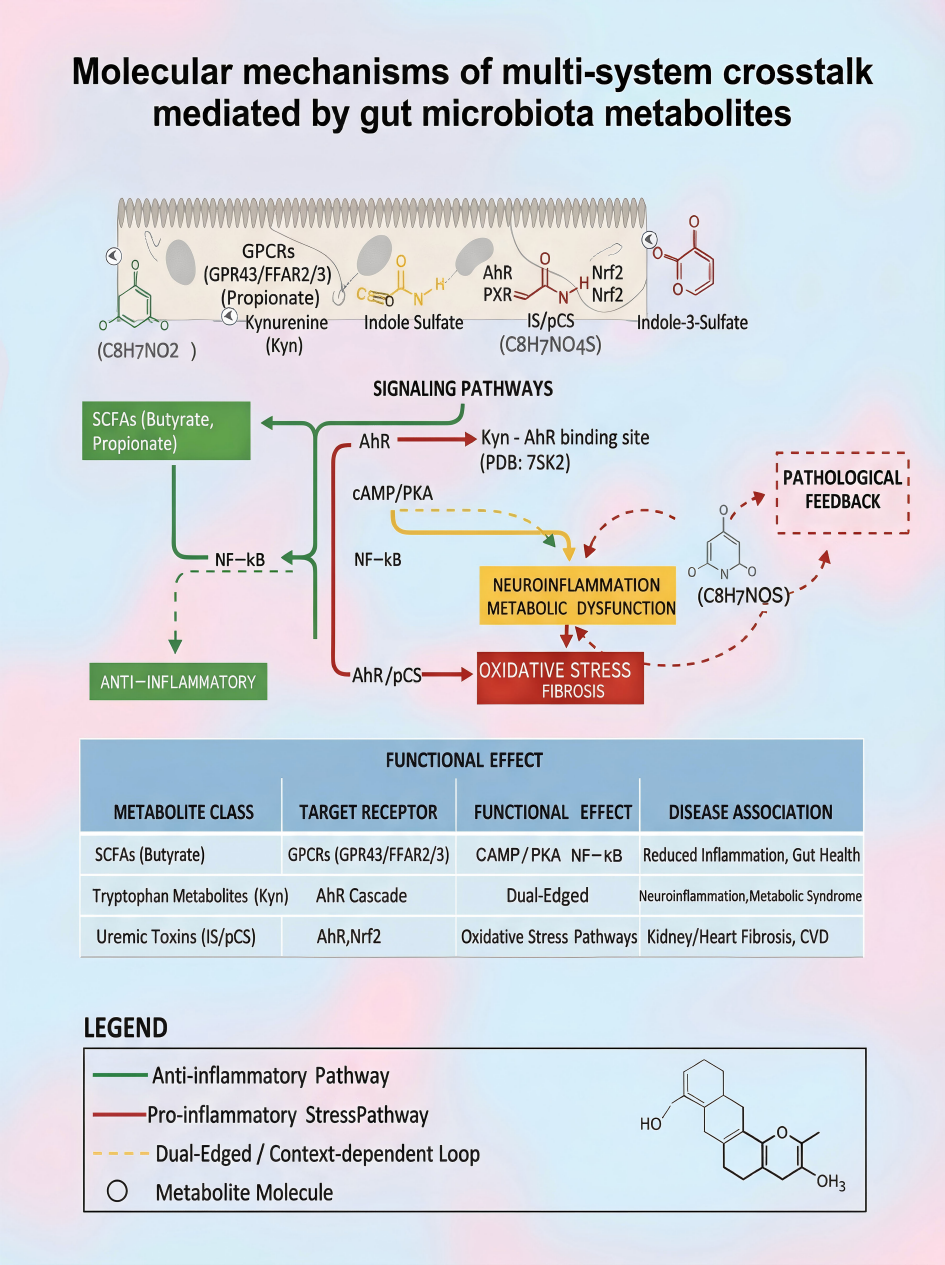
Molecular mechanisms of multi-system crosstalk mediated by gut microbiota metabolites.

Based on these mechanisms, we propose a unified “Microbiota Metabolites–Host Receptors–Multi-Organ Effects” framework (**Figure 3**). This model emphasizes three key processes:(1) Metabolites translocate the intestinal barrier and activate organ-specific signaling via dedicated receptors (e.g., SCFAs → GPCRs; Kynurenine →AhR);(2) Organs communicate through inflammatory factors and neuroendocrine axes, establishing dynamic feedback loops;(3) Disease disrupts metabolic homeostasis, creating pathologic cycles-e.g., indoxyl sulfate (IS) accumulation in CKD further impairs renal function.

Key additions: Ligand-receptor binding: Molecular docking of Kyn-AhR (PDB:7SK2).Signaling annotations: SCFAs→GPCRs↑cAMP/PKA; Kyn→AhR↑IDO1. Crosstalk nodes: Nrf2/NF-κB oxidative stress loop (red lightning).Pathological feedback: Red dashed arrows indicate vicious cycles.

#### 4.4.3 Challenges in clinical translation and future directions

Current research faces three major translational challenges: Individual variability: Genetic polymorphisms (e.g., differential AhR nuclear translocation efficiency) cause heterogeneity in metabolite effects, necessitating SNP genotyping-guided personalized interventions (54). Limitations in dynamic monitoring: Current metabolite assays cannot reflect real-time organ exposure levels; minimally invasive sensors (e.g., implantable intestinal electrodes) offer a promising solution (49, 50). Insufficient interdisciplinary integration: Artificial intelligence can model microbiota–metabolite–host phenotype interactions but requires integrating microbiome, metabolomic, and clinical data (37).

In future research, developing dual inhibitors is crucial for targeting key enzymatic pathways. A promising example is a dual inhibitor of IDO1 and KMO in the kynurenine pathway, which could concurrently reduce neuroinflammation and metabolic imbalances by modulating tryptophan metabolite flux. This strategy may enhance therapeutic efficacy for multi-system disorders like Alzheimer’s and diabetes, leveraging the interconnected gut-brain-axis mechanisms discussed.

## 5 Discussion

### 5.1 Key findings overview

This review synthesizes evidence demonstrating that gut microbiota-derived metabolites—particularly short-chain fatty acids (SCFAs), tryptophan derivatives, and uremic toxins—mediate multi-organ crosstalk along the gut-kidney-heart-brain-endocrine axis. Specifically, SCFAs confer renal and cardioprotective effects through GPR43 activation and NF-κB suppression, whereas tryptophan metabolites exhibit dual roles in neuroprotection and metabolic dysfunction. Conversely, uremic toxins promote oxidative stress and fibrosis, thereby accelerating chronic disease progression. We contextualize these findings within existing literature and clinical implications in the following sections.

### 5.2 Interpretation and context

These findings address clinical dilemmas in cardio-cerebrovascular comorbidities, wherein shared mechanisms such as atherosclerosis and inflammation drive morbidity. Compared to existing literature (1, 2), our results confirm that SCFAs improve renal function (3, 4); however, they highlight inconsistencies arising from methodological heterogeneity. The dual nature of tryptophan metabolites (5, 6) underscores the need for integrated pathways that target both neuroinflammation and metabolic health. Although metabolites like trimethylamine N-oxide (TMAO) show promise as biomarkers (49), translational gaps persist.

### 5.3 Limitations

Our evidence is constrained by substantial heterogeneity (I²=68%) across studies, variations in design (e.g., randomized controlled trials [RCTs] vs. cohort studies), and sample diversity. These limitations may affect the generalizability of findings, necessitating cautious interpretation. Nevertheless, consistent mechanistic insights support the biological plausibility of the observed effects.

### 5.4 Future directions

Future research should develop dynamic, staged intervention models that incorporate real-time biomarker feedback and AI-driven subtyping (49). Cross-disciplinary approaches—integrating neuropsychology and technology (e.g., minimally invasive sensors)—could overcome current limitations. Prioritizing personalized strategies, such as microbiota transplantation-dietary synergies, will bridge the translational gap.

### 5.5 Core conclusions

In summary, gut microbiota metabolites are pivotal in multi-organ crosstalk and offer novel strategies for precision medicine in chronic diseases. Despite challenges, their roles as biomarkers and therapeutic targets hold significant promises for improving patient outcomes through interdisciplinary collaboration.

## References

[1] Alves JLB Costa PCTD Sales LCS Silva Luis CC Bezerra TPT Sousa MLA . Shedding light on the impacts of Spirulina platensis on gut microbiota and related health benefits. Crit Rev Food Sci Nutr. (2025) 65:2062–75. doi: 10.1080/10408398.2024.2323112, PMID: 38420934

[2] Chen N Wu J Wang J Piri N Chen F Xiao T . Short chain fatty acids inhibit endotoxin-induced uveitis and inflammatory responses of retinal astrocytes. Exp Eye Res. (2021) 206:108520. doi: 10.1016/j.exer.2021.108520, PMID: 33617852 PMC8489808

[3] Zhao Y Bi J Yi J Wu X Ma Y Li R . Pectin and homogalacturonan with small molecular mass modulate microbial community and generate high SCFAs via *in vitro* gut fermentation. Carbohydr Polym. (2021) 269:118326. doi: 10.1016/j.carbpol.2021.118326, PMID: 34294338

[4] Zhang Z Yang P Zhao J . Ferulic acid mediates prebiotic responses of cerealderived arabinoxylans on host health. Anim Nutr. (2021) 9:31–8. doi: 10.1016/j.aninu.2021.08.004, PMID: 35949987 PMC9344318

[5] Szrejder M Piwkowska A . Gut microbiome-derived short-chain fatty acids in glomerular protection and modulation of chronic kidney disease progression. Nutrients. (2025) 17:2904. doi: 10.3390/nu17172904, PMID: 40944292 PMC12430357

[6] Lin WY Lin JH Kuo YW Chiang PR Ho HH . Probiotics and their metabolites reduce oxidative stress in middle-aged mice. Curr Microbiol. (2022) 79:104. doi: 10.1007/s00284-022-02783-y, PMID: 35157139 PMC8843923

[7] Xie H Yu S Tang M . Gut microbiota dysbiosis in inflammatory bowel disease: interaction with intestinal barriers and microbiota-targeted treatment options. Front Cell Infect Microbiol. (2025) 15:1608025. doi: 10.3389/fcimb.2025.1608025, PMID: 40654576 PMC12245916

[8] Wang X Xu Y Wang Y Xu Y Tian Y Wang Y . Poricoic acid A protects against high-salt-diet induced renal fibrosis by modulating gut microbiota and SCFA metabolism. Plant Foods Hum Nutr. (2025) 80:115. doi: 10.1007/s11130-025-01356-1, PMID: 40299114

[9] Zhu X Zhao L Lei L Zhu Y Xu J Liu L . Fecal microbiota transplantation ameliorates abdominal obesity through inhibiting microbiota-mediated intestinal barrier damage and inflammation in mice. Microbiol Res. (2024) 282:127654. doi: 10.1016/j.micres.2024.127654, PMID: 38417203

[10] Chen X Xu L Chen Q Su S Zhuang J Qiao D . Polystyrene micro- and nanoparticles exposure induced anxiety-like behaviors, gut microbiota dysbiosis and metabolism disorder in adult mice. Ecotoxicol Environ Saf. (2023) 259:115000. doi: 10.1016/j.ecoenv.2023.115000, PMID: 37210994

[11] Xie X Gu Y Liu Y Shen M Ji J Gao J . An inulin-type fructan from Codonopsis pilosula ameliorates cyclophosphamide-induced immunosuppression and intestinal barrier injury in mice. Int J Biol Macromol. (2025) 310:143312. doi: 10.1016/j.ijbiomac.2025.143312, PMID: 40250123

[12] Rykalo N Riehl L Kress M . The gut microbiome and the brain. Curr Opin Support Palliat Care. (2024) 18:282–91. doi: 10.1097/SPC.0000000000000717, PMID: 39250732

[13] Yan J Kothur K Mohammad S Chung J Patel S Jones HF . CSF neopterin, quinolinic acid and kynurenine/tryptophan ratio are biomarkers of active neuroinflammation. EBioMedicine. (2023) 91:104589. doi: 10.1016/j.ebiom.2023.104589, PMID: 37119734 PMC10165192

[14] Bai Y Zhou X Zhao J Wang Z Ye H Pi Y . Sources of dietary fiber affect the SCFA production and absorption in the hindgut of growing pigs. Front Nutr. (2021) 8:719935. doi: 10.3389/fnut.2021.719935, PMID: 35083261 PMC8784547

[15] Karim MR Morshed MN Iqbal S Mohammad S Mathiyalagan R Yang DC . A network pharmacology and molecular-docking-based approach to identify the probable targets of short-chain fatty-acid-producing microbial metabolites against kidney cancer and inflammation. Biomolecules. (2023) 13:1678. doi: 10.3390/biom13111678, PMID: 38002360 PMC10669250

[16] Rekha K Venkidasamy B Samynathan R Nagella P Rebezov M Khayrullin M . Short-chain fatty acid: An updated review on signaling, metabolism, and therapeutic effects. Crit Rev Food Sci Nutr. (2024) 64:2461–89. doi: 10.1080/10408398.2022.2124231, PMID: 36154353

[17] Foresto-Neto O Ghirotto B Câmara NOS . Renal sensing of bacterial metabolites in the gut-kidney axis. Kidney 360. (2021) 2:1501–9. doi: 10.34067/KID.0000292021, PMID: 35373097 PMC8786145

[18] Hu Z Jia J Su Y Gu Y Cheng B Jiang N . Modified Zexie decoction improves phlegm-dampness type stage I hypertension by regulating the gut-immune-kidney axis. Front Pharmacol. (2025) 16:1578815. doi: 10.3389/fphar.2025.1578815, PMID: 40606611 PMC12215698

[19] Lu PC Hsu CN Lin IC Lo MH Yang MY Tain YL . The association between changes in plasma short-chain fatty acid concentrations and hypertension in children with chronic kidney disease. Front Pediatr. (2020) 8:613641. doi: 10.3389/fped.2020.613641, PMID: 33614542 PMC7890123

[20] Pham QH Bui TVA Sim WS Lim KH Law COK Tan W . Daily oral administration of probiotics engineered to constantly secrete short-chain fatty acids effectively prevents myocardial injury from subsequent ischaemic heart disease. Cardiovasc Res. (2024) 120:1737–51. doi: 10.1093/cvr/cvae128, PMID: 38850165 PMC11587561

[21] Wang X Dong Y Huang R Wang F Xie J Liu H . The role of short-chain fatty acids in myocardial ischemia-reperfusion injury. Curr Nutr Rep. (2024) 13:701–8. doi: 10.1007/s13668-024-00564-6 PMC11489193 39110372

[22] Poll BG Xu J Jun S Sanchez J Zaidman NA He X . Acetate, a short-chain fatty acid, acutely lowers heart rate and cardiac contractility along with blood pressure. J Pharmacol Exp Ther. (2021) 377:39–50. doi: 10.1124/jpet.120.000187, PMID: 33414131 PMC7985618

[23] Hu T Wu Q Yao Q Jiang K Yu J Tang Q . Short-chain fatty acid metabolism and multiple effects on cardiovascular diseases. Ageing Res Rev. (2022) 81:101706. doi: 10.1016/j.arr.2022.101706, PMID: 35932976

[24] Rivera K Gonzalez L Bravo L Manjarres L Andia ME . The gut-heart axis: molecular perspectives and implications for myocardial infarction. Int J Mol Sci. (2024) 25:12465. doi: 10.3390/ijms252212465, PMID: 39596530 PMC11595032

[25] Furukawa N Kobayashi M Ito M Matsui H Ohashi K Murohara T . Soy protein b-conglycinin ameliorates pressure overload-induced heart failure by increasing short-chain fatty acid (SCFA)-producing gut microbiota and intestinal SCFAs. Clin Nutr. (2024) 43:124–37. doi: 10.1016/j.clnu.2024.09.045, PMID: 39447394

[26] Wimmer MI Bartolomaeus H Anandakumar H Chen CY Vecera V Kedziora S . Metformin modulates microbiota and improves blood pressure and cardiac remodeling in a rat model of hypertension. Acta Physiol (Oxf). (2024) 240:e14226. doi: 10.1111/apha.14226, PMID: 39253815

[27] Rosell-Cardona C Leigh SJ Knox E Tirelli E Lyte JM Goodson MS . Acute stress-induced alterations in short-chain fatty acids: Implications for the intestinal and blood brain barriers. Brain Behav Immun Health. (2025) 46:100992. doi: 10.1016/j.bbih.2025.100992, PMID: 40510181 PMC12159890

[28] Liou CW Yao TH Wu WL . Intracerebroventricular delivery of gut-derived microbial metabolites in freely moving mice. J Vis Exp. (2022) 184. doi: 10.3791/63972, PMID: 35723471

[29] Zhang Q Li H Yin S Xiao F Gong C Zhou J . Changes in short-chain fatty acids affect brain development in mice with early life antibiotic-induced dysbacteriosis. Transl Pediatr. (2024) 13:1312–26. doi: 10.21037/tp-24-128, PMID: 39263295 PMC11384438

[30] Li C Yao J Yang C Yu S Yang Z Wang L . Gut microbiota-derived short chain fatty acids act as mediators of the gut-liver-brain axis. Metab Brain Dis. (2025) 40:122. doi: 10.1007/s11011-025-01554-5, PMID: 39921774

[31] Wijdeveld M Schrantee A Azor JT van Baarzel F van Duinkerken E Nieuwdorp M . Intestinal short-chain fatty acid turnover is not associated with resting state functional connectivity in mesolimbic dopaminergic network in healthy adults. NeuroImage Rep. (2025) 5:100285. doi: 10.1016/j.ynirp.2025.100285, PMID: 40893427 PMC12398794

[32] Liang Y Xie S He Y Xu M Qiao X Zhu Y . Kynurenine pathway metabolites as biomarkers in alzheimer’s disease. Dis Markers. (2022), 9484217. doi: 10.1155/2022/9484217, PMID: 35096208 PMC8791723

[33] Cortés Malagón EM López Ornelas A Olvera Gómez I Bonilla Delgado J . The kynurenine pathway, aryl hydrocarbon receptor, and Alzheimer’s disease. Brain Sci. (2024) 14:950. doi: 10.3390/brainsci14090950, PMID: 39335444 PMC11429728

[34] Kearns R . The kynurenine pathway in gut permeability and inflammation. Inflammation. (2025) 48:1063–77. doi: 10.1007/s10753-024-02135-x, PMID: 39256304 PMC12234587

[35] Arto C Rusu EC Clavero-Mestres H Barrientos-Riosalido A Bertran L Mahmoudian R . Metabolic profiling of tryptophan pathways: Implications for obesity and metabolic dysfunction-associated steatotic liver disease. Eur J Clin Invest. (2024) 54:e14279. doi: 10.1111/eci.14279, PMID: 38940215

[36] Zhao P Chen Y Zhou S Li F . Microbial modulation of tryptophan metabolism links gut microbiota to disease and its treatment. Pharmacol Res. (2025) 219:107896. doi: 10.1016/j.phrs.2025.107896, PMID: 40763909

[37] Chen Z Xiao C Zhang J Jian S Li P Lin J . The impact of diet on the colonization of beneficial microbes from an ecological perspective. J Agric Food Chem. (2025) 73:10069–92. doi: 10.1021/acs.jafc.5c02086, PMID: 40234746

[38] Chen X Xu D Yu J Song XJ Li X Cui YL . Tryptophan metabolism disordertriggered diseases, mechanisms, and therapeutic strategies: A scientometric review. Nutrients. (2024) 16:3380. doi: 10.3390/nu16193380, PMID: 39408347 PMC11478743

[39] Fellendorf FT Gostner JM Lenger M Platzer M Birner A Maget A . Tryptophan metabolism in bipolar disorder in a longitudinal setting. Antioxid (Basel). (2021) 10:1795. doi: 10.3390/antiox10111795, PMID: 34829665 PMC8615217

[40] Sato E Hosomi K Sekimoto A Mishima E Oe Y Saigusa D . Effects of the oral adsorbent AST-120 on fecal p-cresol and indole levels and on the gut microbiota composition. Biochem Biophys Res Commun. (2020) 525:773–9. doi: 10.1016/j.bbrc.2020.02.141, PMID: 32147096

[41] Rocchetti MT Cosola C Ranieri E Gesualdo L . Protein-bound uremic toxins and immunity. Methods Mol Biol. (2020) 2325:215–27. doi: 10.1007/978-1-0716-1507-2_15, PMID: 34053061

[42] Lano G Laforêt M Von Kotze C Perrin J Addi T Brunet P . Aryl hydrocarbon receptor activation and tissue factor induction by fluid shear stress and indoxyl sulfate in endothelial cells. Int J Mol Sci. (2020) 21:2392. doi: 10.3390/ijms21072392, PMID: 32244284 PMC7178278

[43] Croci S D’Apolito LI Gasperi V Catani MV Savini I . Dietary strategies for management of metabolic syndrome: role of gut microbiota metabolites. Nutrients. (2021) 13:1389. doi: 10.3390/nu13051389, PMID: 33919016 PMC8142993

[44] Guthrie L Spencer SP Perelman D Van Treuren W Han S Yu FB . Impact of a 7-day homogeneous diet on interpersonal variation in human gut microbiomes and metabolomes. Cell Host Microbe. (2022) 30:863–874.e4. doi: 10.1016/j.chom.2022.05.003, PMID: 35643079 PMC9296065

[45] Rysz J Franczyk B Ławiński J Olszewski R Ciałkowska-Rysz A Gluba-Brzózka A . The impact of CKD on uremic toxins and gut microbiota. Toxins (Basel). (2021) 13:252. doi: 10.3390/toxins13040252, PMID: 33807343 PMC8067083

[46] Yan K Sun X Wang X Zheng J Yu H . Gut microbiota and metabolites: biomarkers and therapeutic targets for diabetes mellitus and its complications. Nutrients. (2025) 17:2603. doi: 10.3390/nu17162603, PMID: 40871631 PMC12389361

[47] Wagner CA Massy ZA Capasso G Mattace-Raso F Pepin M Bobot M . Translational research on cognitive impairment in chronic kidney disease. Nephrol Dial Transpl. (2025) 40:621–31. doi: 10.1093/ndt/gfae229, PMID: 39400744

[48] Shen Y Yu C . The bone-vascular axis: A key player in chronic kidney disease associated vascular calcification. Kidney Dis (Basel). (2024) 10:545–57. doi: 10.1159/000541280, PMID: 39664335 PMC11631106

[49] Chung SJ Rim JH Ji D Lee S Yoo HS Jung JH . Gut microbiota-derived metabolite trimethylamine N-oxide as a biomarker in early Parkinson’s disease. Nutrition. (2021) 83:111090. doi: 10.1016/j.nut.2020.111090, PMID: 33418492

[50] Luo J Luo M Kaminga AC Wei J Dai W Peng Y . Integrative metabolomics highlights gut microbiota metabolites as novel NAFLD-related candidate biomarkers in children. Microbiol Spectr. (2024) 12:e0523022. doi: 10.1128/spectrum.05230-22, PMID: 38445874 PMC10986516

[51] Tonch-Cerbu AK Boicean AG Stoia OM Teodoru M . Gut microbiota-derived metabolites in atherosclerosis: pathways, biomarkers, and targets. Int J Mol Sci. (2025) 26:8488. doi: 10.3390/ijms26178488, PMID: 40943409 PMC12429262

[52] Dasriya VL Samtiya M Ranveer S Dhillon HS Devi N Sharma V . Modulation of gut-microbiota through probiotics and dietary interventions to improve host health. J Sci Food Agric. (2024) 104:6359–75. doi: 10.1002/jsfa.13370, PMID: 38334314

[53] Dong Y Gai Z Han M Xu J Zou K . Reduction in serum concentrations of uremic toxins driven by bifidobacterium longum subsp. Longum BL21 is associated with gut microbiota changes in a rat model of chronic kidney disease. Probiotics Antimicrob Proteins. (2025) 17:1893–904. doi: 10.1007/s12602-024-10293-5, PMID: 38829564 PMC12405026

[54] Gibbons SM Gurry T Lampe JW Chakrabarti A Dam V Everard A . Perspective: leveraging the gut microbiota to predict personalized responses to dietary, prebiotic, and probiotic interventions. Adv Nutr. (2022) 13:1450–61. doi: 10.1093/advances/nmac075, PMID: 35776947 PMC9526856

[55] Clerici L Bottari D Bottari B . Gut microbiome, diet and depression: literature review of microbiological, nutritional and neuroscientific aspects. Curr Nutr Rep. (2025) 14:30. doi: 10.1007/s13668-025-00619-2, PMID: 39928205 PMC11811453

[56] Lai H Li Y He Y Chen F Mi B Li J . Effects of dietary fibers or probiotics on functional constipation symptoms and roles of gut microbiota: a double-blinded randomized placebo trial. Gut Microbes. (2023) 15:2197837. doi: 10.1080/19490976.2023.2197837, PMID: 37078654 PMC10120550

